# Articaine: dental practitioner use, basis of perception and evidence-based dentistry—a cross-sectional study

**DOI:** 10.1038/s41405-022-00113-9

**Published:** 2022-07-04

**Authors:** Erica Martin, Andrew Lee, Ernest Jennings

**Affiliations:** James Cook University, 14-88 McGregor Road, Smithfield, QLD 4878 Australia

**Keywords:** Dental anaesthesia, Special care dentistry

## Abstract

**Background:**

Limited data exist on dental practitioner use and perceptions of articaine. This study is a cross-sectional survey of dental practitioners from January, 2021 to ascertain the extent of their use of the dental local anaesthetic, articaine, the basis of their perceptions about articaine and whether current practices are in line with recent evidence regarding articaine safety and efficacy.

**Method:**

An anonymous survey was designed using the SAP Qualtrics Core XM software platform and a survey link was disseminated from December 2020 to January 2021 via social media. The survey was designed as a five minute, anonymous, online questionnaire including a plain language information sheet, request for participant consent and 14 questions. Data were entered onto a Microsoft™ Excel spreadsheet and analysed qualitatively, isolating the answers into recurrent themes.

**Results:**

Sixty percent of the surveyed dental practitioner used articaine as their preferred dental anaesthetic. Twenty-three percent of the dental practitioner surveyed used articaine for all of their dental procedures including inferior alveolar nerve blocks, while 40% of respondents used articaine for all their dental procedures except inferior alveolar nerve blocks. The predominant basis of dental practitioner uses and perception of articaine were their countries dental guidelines.

**Conclusion:**

Despite the latest findings that articaine is as safe and more efficacious as lidocaine for all routine dental treatment, 40% of survey respondents avoided articaine use for inferior alveolar blocks. Our study recognises a discrepancy between reported clinical practice and current research evidence. Further research and clarifications are needed to achieve ubiquitous practice of evidence-based dentistry.

## Introduction

Articaine is an amide local anaesthetic (LA) used routinely in dental practice since its clinical release in 1976 [1, 2]. Prior to articaine’s release, lidocaine was the most commonly used dental LA worldwide [3]. In 1995, Haas and Lennon released a review suggesting a link between articaine use and increased incidence of lingual nerve paraesthesia [4]. In addition, in 2009 and 2010, further reviews involving the same researcher revisited the association and further postulated a link between 4% LA solutions and increased incidence of lingual nerve paraesthesia [5, 6]. These reviews approximated the occurrence of LA-related paraesthesia to be 1 in 609,000 in 2009 [5]. The rates were then revised to 1 in 4,159,848 in 2010 [6].

Systematic reviews and meta-analysis are considered the highest, most robust analysis of clinical efficacy across multiple trials [7, 8]. Multiple systematic reviews have been conducted on articaine efficacy and safety from 2010 to the present, none of them, nor any of the randomised controlled trials analysed by the reviews reported incidence of permanent nerve paraesthesia following articaine use [9–12].

The latest articaine systematic review with meta-analyses conducted by Martin et al. in 2021 stated that articaine is a safe and efficacious LA for all routine dental treatment [12]. None of the participants in the 14 randomised controlled trials reported any major LA-related adverse effects. The results from Martin et al.’s latest systematic review are consistent with older reviews of articaine efficacy and safety [9–11]. Despite copious evidence corroborating articaine safety and efficacy, articaine still bears the stigma from the earlier review results that may have been subject to bias and conducted with less-than robust research techniques.

## Background

Limited data exist on dental practitioner use and perceptions of articaine, especially related to articaine use for the standard inferior alveolar nerve block (IANB). Yapp et al. surveyed Australian Dental Association members in 2010 to ascertain the Australian dental practitioner use of articaine, the reason for their choice of LA and their level of education [13]. Their survey found that the majority of Australian dental professionals used articaine and cited scientific literature, professional education courses and peer reports as the main influences behind their choice of LA. The study further detailed that one third of respondents used articaine for all procedures except the IANB [13]. In 2010, systematic reviews and randomised controlled trials existed finding articaine to be, equal to or more efficacious, and as safe as lidocaine [9]. Despite the research dictates in 2010, dental practitioners remained cautious in their use articaine for IANBs.

Our cross-sectional study follows a decade on from Yapp et al.’s research to determine current dental practitioner use of articaine, the basis of their perceptions and if they are practicing evidence-based dentistry in 2021. Evidence-based dentistry has been defined by the Australian Dental Association as an approach to dental practice that requires integration of systematic assessment and clinically relevant scientific evidence with dental practitioner clinical experience expertise and patient’s health perspectives [14].

The aim of this cross-sectional study was to ascertain if dental practitioners as of January 2021 are enacting evidence-based dentistry. The research process involved two steps. Firstly, the gathering of survey data about dental practitioner use of articaine, their perceptions of articaine and the basis of their perceptions about articaine. Secondly, determining if the survey data results align with the latest robust evidence about the safety and efficacy of articaine in routine dentistry. Any discrepancy in evidence-based practice indicates a need for further research to clarify any misconceptions about articaine use for all routine dental treatment.

### Methodology

The research project was approved by the James Cook University Human Research Ethics Committee approval number H8223.

The authors used the SPIDER qualitative/mixed-methods strategy tool [15] to outline the research questions:

- Sample—dental practitioners.
- Phenomenon of Interest—use of and perceptions about dental local anaesthetic and basis of their perceptions of dental LA.
- Design—survey.
- Evaluation—experiences and perceptions.
- Research type—qualitative/quantitative.

Research question:

- What percentage of dental practitioners use articaine?
- What are dental practitioner perceptions about the safety and efficacy of articaine compared to other dental LAs?
- What are the factors that influence dental practitioner perceptions about articaine and dental LAs?

An anonymous survey was designed using the SAP Qualtrics Core XM software platform. The survey questions were piloted and, subsequently, reviewed and validated by a group of dental professionals comprising of practising dentists, dental specialists and university-affiliated professors of clinical dentistry. Survey questions were revised according to the recommendations advised by the group [16].

The survey link was disseminated from December 2020 to January 2021 via social media. The Qualtrics survey link was posted on three private Facebook pages dedicated to dental professionals around the world. The reach between the three Facebook groups, at the time, was ~80,000 members, with possible overlap in membership between the groups. Given the international membership and accessibility of the private dental Facebook groups, the survey was accessible to dental professionals globally.

Online surveys are a timely, far-reaching and cost-effective method of data collection [17], and participants are more likely to give honest answers if they do not have to disclose personal details [18]. Social media has become an effective avenue for researchers to increase their global reach. In addition, considering the current global climate, the move to online communication is the most COVID-safe data collection strategy [18].

Our survey was designed as a five minute, anonymous, online questionnaire including a plain language information sheet (Appendix 1), request for participant consent and 14 questions (Appendix 2). The survey questions consisted of mixed multiple-choice answers and text boxes that requested information about participant:

- Demographics—practice field, sector of practice and country of registration.
- Dental local anaesthetic use and preference.
- Use of articaine in dental practice and for inferior alveolar nerve blocks.
- View of articaine safety and efficacy.
- Basis of perceptions of articaine use in routine dental practice.
- Views of articaine compared to lidocaine in terms of safety and efficacy.
- Experience of adverse reactions on any LA following inferior alveolar nerve blocks. If any:
  - Which LA was used.
  - What adverse reactions were experienced by the patient.
  - Any change in clinical practice following the experience.
- Any further information they would like to share about LA-related adverse events.

Data was extracted onto a Microsoft™ Excel spreadsheet and analysed qualitatively, isolating the answers into recurrent themes.

## Results

A total of 325 completed surveys were returned out of 358 respondents. All respondents who completed the survey consented to participating in the study. The remaining 33 surveys were incomplete or blank, possibly due to connectivity issues, hard/software issues or human factors.

Three-quarters of survey respondents were Australian-registered general dentists working in the private and public sectors (Table 1), with the United Kingdom having the second most survey respondents at seven percent.

**Table 1:** Demographics of survey participants.

Profession	Sector of practice	Country of registration
General dentist 83%	Private 69%	Australia 76%
Dental specialists 7.4%	Public 18%	United Kingdom 7%
Oral Health Therapists 4%	Education 8%	New Zealand 3%
Dental students 3%	Research 2%	Canada 2%
Dental Therapists 1%	Other 3%	U.S.A. 2%
Dental Hygienists 1%		Singapore 0.5%
Post graduate student 0.5%		Pakistan 0.5%
		Scotland 0.5%
		Malaysia 0.5%
		Ireland 0.5%
		India 0.3%
		Romania 0.3%
		Trindad Tobego 0.3%
		Slovenia 0.3%
		Israel 0.3%
		Switzerland 0.3%
		Hungary 0.3%
		Norway 0.3%
		Germany 0.3%
		Wales 0.3%
		Barbados 0.3%

### What percentage of dental practitioners use articaine?

Sixty percent of the dental practitioner surveyed used articaine as their preferred dental anaesthetic. Thirty-five percent preferred lidocaine, 2% preferred mepivacaine and 1% preferred prilocaine as their primary dental anaesthetic.

### What are dental practitioner perceptions about the safety and efficacy of articaine compared to other dental LAs?

Twenty-three percent of the dental practitioner surveyed use articaine for all of their dental procedures including IANB. Forty percent of respondents use articaine for all their dental procedures except those requiring IANB. Other variations of this answer were:

- Mainly use articaine except for pregnant women.
- Mainly use articaine except for children under five years of age.
- Mainly use articaine except for when contraindicated (no further details given).

Fifty-six percent of dental practitioners surveyed felt confident using articaine for all routine dental procedures, 38% felt confident using articaine for some dental procedures, and 2% did not feel confident using articaine for any dental procedures.

Regarding articaine safety and efficacy compared to lidocaine:

- Forty-six percent of survey respondent felt articaine to be as safe and more efficacious compared to lidocaine.
- Thirty-one percent felt articaine to be more efficacious, but less safe than lidocaine.
- Eighteen percent felt articaine to be as safe and efficacious as lidocaine.
- Two respondents felt that articaine is not safe to be used as a dental LA.

### What are the factors that influence their perceptions?

The main basis of dental practitioner use and perception according to our survey were: their countries dental guidelines (25%), ongoing professional development courses (20%), their university teachings (16%), their own research (15%), advice from dental colleagues (14%) and advice from their dental mentors (7%). Other sources listed were indemnity insurer advice, experience, and manufacture’s advice.

### What LA-related adverse effects have dental practitioners experienced following administering of an inferior alveolar nerve block?

Of the 325 respondents, 13% had experienced a patient with LA-related adverse effects more than one day after the administration of a standard IANB.

The dental LA’s which caused these adverse effects were lidocaine (47%), articaine (47%), prilocaine (4%) and mepivicaine (2%).

The adverse effects experienced: paraesthesia (38%); palpitations, anxiety, shaking (13%); swelling and bruising (11%); trismus (10%), haematoma (7%), neuropathy (6%), palsy (4%), vision changes (3%) and syncope (1%). Other adverse effects (4%) included breathing and swallowing difficulties, numbness under the eye, pain lasting over two weeks in the injection site and grand mal seizures. The breakdown of adverse effects by LA can be found in Table 2.

**Table 2:** Adverse effects by dental anaesthetic type.

Adverse Effects	Articaine	Lidocaine	Prilocaine	Mepivicaine	Other	Total
Paraesthesia	15	10		1	1	27
Neuropathy	3	1				4
Trismus		6			1	7
Haematoma		3	1			4
Palsy	1	2				3
Palpitations/anxiety/shaking	2	7				9
Syncope		1				1
Breathing/swallowing difficulty		1				1
Swelling/bruising	1	6	1	1		9
Pain >2 week	1					1
Numbness under the eye	1					1
Vision changes	1				1	2

Sixty-eight of the dental practitioners surveyed who experienced LA-related adverse effects in their patients did not change their clinical procedure following the experience. Fourteen percent did not repeat the procedure using the LA that caused the adverse effects. Five percent stopped using the LA associated with the adverse effect. Other clinical changes (12%) were: changing to a smaller gauge needle, practicing their mandibular block technique, stopping administering LA when the patient experiences unusual pain, and stopping using the LA for IANB.

Respondents were asked if they wanted to share any further information about their adverse effect experiences. The general themes were:

- The need to review their injection technique and not blame the dental LA for the adverse reaction experienced by their patient.
- The awareness of needle technique when the patient feels an electric shock during needle insertion and not to blame the LA for the adverse effect.
- Only one adverse effect in 20 years of practice, and in another case, in 15 years of practice, only one case of paraesthesia, and, another case with 50 years of experience has only experienced minor reactions with one case of prolonged paraesthesia that resolved after a year.
- The temporary nature of the adverse effects.

Two further experiences shared were of:

- Lidocaine used for maxillary infiltration that caused the patient to have grand mal seizures and they were in hospital for four days with no history of epilepsy.
- Lingual paraesthesia following using articaine for inferior alveolar nerve blocks, but all reported cases resolved.

## Discussion

### Survey results 2010 vs. 2021

The majority of dental practitioners who responded to our 2021 survey used articaine as their preferred dental LA. Our study data corroborate the results published by a similar 2010 study [13]. In contrast, our study found that 40% of survey respondents avoided articaine use for IANB, an increase of 10% from the 2010 study.

The authors of the current study published a 2021 systematic review of randomised controlled trials ascertaining the safety and efficacy of articaine which concluded that articaine is a safe and efficacious dental LA for all routine dental procedures. Thus, the current survey study reveals a potential discrepancy in evidence-based dental practice related to dental LA use and the underlying factors should be addressed.

### Factors influencing practitioner perceptions about dental LA

Our study aimed to ascertain the basis for dental practitioner perceptions for their use of dental LA, this includes the factors that influence their LA choice for various dental procedures. The top three factors determining dental practitioner perceptions of dental LA were: their countries dental guidelines, continuing professional development courses and their university teachings (Table 3). The most common basis that influenced dental LA choice was country of registration dental guidelines.

**Table 3:** Factors affecting practitioner perception of dental local anaesthetics.

Factors^a^	Other factors	x/325	%
Country of registration guidelines		210	25%
Professional courses		157	20%
Dental degree course		132	16%
Own research		125	15%
Dental colleagues		110	14%
Mentors		58	7%
Other		27	3%
	Evidence-based studies	1	
	Indemnity Insurer	1	
	Experience	8	
	Litigation experience	1	
	Manufacturer’s instructions	2	

^a^Survey participants were able to choose multiple answers.

In addition to the influences mentioned above, some of the respondents avoided articaine use in pregnant women, children under the age of five and where contraindicated (no specifics given).

### Articaine use in children under 4 years of age

A 2020 randomised controlled trial assessed articaine’s efficacy and safety in children under four years of age [19]. One hundred and eighty-four children aged 36–47 months were anaesthetised with either articaine or lidocaine for dental pulpotomies. The study concluded that children administered articaine experienced less pain during treatment and there was no statistical difference detected between the two LAs regarding post-operative complications [19].

The most recent systematic review and meta-analysis reviewing articaine and lidocaine in children’s dentistry concluded that there was no difference in the occurrence of adverse events between articaine and lidocaine following treatment in paediatric patients [20]. The review only included studies of children aged 5–16.

Ezzeldin et al. published a 2020 review of United Kingdom paediatric specialist views of the use of articaine in paediatric dentistry [21]. The review concluded that participants of the study reported more adverse effects with lidocaine than with articaine. Also, that use of articaine in paediatric dentistry is common, but limited evidence exists to support its use for children under four years of age [21]. More research is needed on the subject.

### Articaine and pregnancy

Scarce research exists on the use of dental LA on pregnant women; therefore, this section will focus on articaine pharmacology and the few in-vitro studies of dental LA on human and rodent neuronal cells.

Articaine is an amide anaesthetic containing an ester group and a thiophene ring [1, 22, 23]. These features are an integral part of articaine’s LA efficacy [24] and rapid plasma hydrolysis [2, 3, 9]. The thiophene ring facilitates articaine diffusion through the nerve cell membrane and into the soft tissue [2, 3, 9]. The ester group allows for rapid plasma hydrolysis [13, 23].

This explains articaine’s shorter half-life of 20–30 mins compared to the half-life of lidocaine and the other amide LAs that require 90–120 mins for hepatic clearance [1, 2, 9, 23]. In addition, 90% of articaine is broken down into its inert form, articainic acid in the plasma sparing liver biotransformation [1]. Articaine’s shorter half-life becomes relevant during lengthy procedures where additional LA needs to be administered or if attempting to minimise systemic or liver toxicity [25].

A 2015 preclinical, in vitro study of dental anaesthetic reported that the studied LAs, lidocaine, articaine, mepivacaine, bupivacaine, prilocaine and ropivicane, all induced human neuroblastoma cell death in increased concentration [26]. The study concluded that articaine and ropivacaine were the least neurotoxic. Lidocaine, mepivicaine and prilocaine were of medium neurotoxicity, and bupivicaine was found to be the most neurotoxic. Neurotoxicity was defined in this study as LD_50_ or the amount needed to achieve 50% cell death [26].

Potocnik et al.’s 2006 study of LA and rodent nerve cells found that 4% articaine was the most effective at blocking nerve conduction of action potentials compared to 2% lidocaine and 3% mepivacaine [27].

### Practitioner perceptions about articaine

Half the survey respondents felt confident using articaine for all their routine dental procedures, with 38% feeling confident to use articaine for some procedures and two respondents (0.0006%) not confident to use articaine at all. With strong data corroborating articaine’s safety, the question should be asked: what factors have influenced the practitioners who do not feel confident using articaine for some or any dental procedures?

### Articaine vs. lidocaine

Three-quarters of the survey respondents felt that articaine is more efficacious a dental LA than lidocaine, which is in line with the conclusions from current research about articaine efficacy. Half of the respondents felt that articaine is equally as safe to use as a dental LA as lidocaine, which is also in line with the current research about articaine safety.

Discrepancies between dental evidence-based practice and current clinical practice about articaine safety were found in our study, with one third of all respondents feeling articaine to be less safe than lidocaine. Another question arises: what factors have influenced practitioners to believe that articaine is less safe than lidocaine?

As outlined in Table 3, the major factors contributing to practitioner perception of dental LA use were countries dental guidelines, continuing professional development courses, university teachings, advice from colleagues, advice from mentors, advice from indemnity insurers and personal experience.

### Adverse reaction experience—standard inferior alveolar nerve block

Thirteen percent of all survey respondents had experienced a patient with LA-related adverse effect following administration of a standard IANB. Of these adverse effects, the majority occurred with use of lidocaine (47%) and articaine (47%). Forty percent of these patients who had suffered LA-related adverse effect experienced nerve paraesthesia as one of the adverse effect. In other words, 0.8% of total respondents had patients who experienced nerve paraesthesia after administration of an IANB. The specific nerve affected was not detailed.

Two respondents had two separate occasions of patients experiencing paraesthesia after an IANB, one with lidocaine and one with articaine. Eight respondents had patients experience paraesthesia in addition to multiple other adverse effects such as palsy, trismus, palpitations, anxiety, shakes, vision changes, swelling and bruising. These simultaneous, multiple adverse reactions following administration of an IANB may have us query what other factors could have caused the reactions other than a reaction to the dental LA? Some hypothesised reasons are: incorrect injection technique, soft tissue trauma, depositing LA too rapidly into the injection site, injection of LA into a blood vessel and blood pooling following injection withdrawal.

Seventeen of the twenty-six (0.05%) adverse effects respondents had patients who only experienced paraesthesia after the IANB with no other adverse effects. Of these, 13 were administered articaine and four were administered lidocaine.

The respondents who had these experiences were queried about how the experience affected their future treatment decisions. Three-quarters answered that they made no changes to their clinical procedures after having an adverse experience following their IANB administration. The remaining respondents answered that they either: practiced or studied to improve their IANB technique, changed their LA type for IANBs, started using smaller gauge needles for their IANBs or made changes to their IANB technique.

The respondents who had these experiences were also given an opportunity to add their thoughts about their experiences. Three recurring themes emerged from this query: adverse effects compared to years of dental experience, awareness that needle technique could be a cause of the adverse effects and the temporary nature of the adverse effects they experienced. Three respondents commented that they had only observed one case of temporary paraesthesia or only minor LA reactions in their 15, 20 and 50 years of dental practice, adding that LA-related adverse effects are rare in their experience. The longest serving practitioner had only experienced one case of LA-related paraesthesia in their 50 years of dental practice and that case resolved after 1 year.

Other personal experiences were a patient who had a grand mal seizure and was hospitalised for four days after lidocaine was used for a maxillary infiltration, and another practitioner who had multiple experiences of patients with lingual paraesthesia after using articaine for IANBs, of which all resolved within a short time period.

### Perception of risk

The overall occurrence of IANB, LA-related adverse effects in our study was 13%. The occurrence of paraesthesia was 0.08%. The risk of LA-related paraesthesia in previous studies has been approximated to be between 1 in 726,000 and 1 in 785,000 [28]. One of the given reasons why robust studies are not available associating nerve paraesthesia to articaine IANBs is because the incidence is so rare [4, 5]. Dental researchers continue to debate about the possible and probable causes of nerve paraesthesia following IANBs, especially about its increased affectation of the lingual nerve [6]. Suggested causes by these researchers are: direct needle trauma, intra-neural haematoma formation, fascicular pattern and LA toxicity [28].

### Limitations

Sample representativeness may be questionable as non-technology savvy and offline dental practitioners were not included, and participants were not globally representative. Seventy-five percent of participants were dental practitioners registered in Australia. The cost of dental LA could be a factor that influences dental practitioner choice of LA, but this was not queried in the survey. In Australia, articaine costs more than lidocaine.

Despite surveys being cost-effective, time-effective and convenient, low response rates may result in non-response bias, unclear responses that cannot be clarified and limited sampling may impact population generalisability [29].

## Conclusion

Our research found that the majority (60%) of queried dental practitioners used articaine as their preferred dental LA. Despite the latest findings that articaine is as safe and more efficacious as lidocaine for all dental treatment, 40% of surveyed dental practitioners avoided articaine use for inferior alveolar blocks citing their countries dental guidelines, ongoing professional development courses and their university teachings as the main factors that influenced their perceptions about dental LA. Our study recognises a discrepancy between reported clinical practice and current research evidence. Further research and clarifications are needed to achieve ubiquitous practice of evidence-based dentistry.

## APPENDIX 1

**Survey Information Sheet**

Project: Articaine in dentistry: dental practitioner perception

Researchers:

Dr Erica Martin

Dr Ernest Jennings

Prof Alan Nimmo

A/Prof Andrew Lee

Introduction

We would like to invite you to participate in an online survey for our dental research project investigating dental practitioner use of articaine and perception of articaine’s safety and efficacy in routine dental procedures.

This project has been approved by the Research Ethics Committee at James Cook University (HREC #H8223).

What is the aim of the research?

The aim of this research is to evaluate the use of articaine in routine dental practice and dental practitioner perception of the safety and efficacy of the local anaesthetic, articaine, for use in all routine dental procedures, and the basis for the perception.

What will participants be asked to do?

Participants who choose to participate will remain anonymous and be asked to answer 12 questions about their dental local anaesthetic choices and views. The answers will help us understand the prevailing views of articaine in dentistry and ascertain the basis for these views.

How long will the survey take to complete?

The survey consists of 12 questions and should take ~5–7 min to complete

Can participants withdraw from the study at any time?

Participants can withdraw from participation in the survey at any time during the survey until they choose to submit their data. After submission, being de-identified, data cannot be withdrawn.

What are the possible risks?

There are no risks involved in participating in this research.

Will participants get access to the results?

Participants will be able to access results of this survey through publication of the research in the dental literature

What will happen to participant information?

Participant answers are anonymous with no personal association or possibility of identification. The researchers will have no means of identifying the responses. All collected data will be kept confidential and securely stored locked with password protection according to JCU Code for the Responsible Conduct of Research. The JCU Code is adapted from the National Code (2007). Section 2.

Are there any potential conflicts of interest?

Dr Erica Martin is a dental practitioner in Australia and is conducting her Master of Philosophy (Health) at James Cook University in Cairns. She has no conflicts of interest.

Where can participants get further information?

Please contact Dr Erica Martin for further information: erica.martin@jcu.edu.au

Who to contact about concerns/complaints about the project?

Human Ethics Officer, Research Office, James Cook University, Townsville, Qld, 4811

Email: ethics@jcu.edu.au Ph: (07) 4781 5011 Fax: (07) 4781 5521

## APPENDIX 2

**Survey questions**

Articaine in dentistry: dental practitioner perception

Do you consent to participate in this anonymous survey?

- Yes, I have read the informed consent information above
- No

Survey questions

1. My profession:
  - General dentist
  - Dental specialist (Specialty___________)
  - Oral health therapist
  - Dental therapist
  - Dental hygienist
  - Dental student
  - Other ______________
2. In which country are you registered as a dental practitioner? ______________
3. In what sector do you primarily practice? Please tick applicable
  - Private
  - Public
  - Education
  - Research
  - Other ________
4. Which dental local anaesthetic would you use for routine (non-surgical) dental procedures, if you had no financial or practice constraints?
  - Lidocaine
  - Articaine
  - Mepivacaine
  - Prilocaine
  - Other__________
5. Which dental local anaesthetic do you currently use for routine (non-surgical) dental procedures? Please tick all applicable:
  - Lidocaine
  - Articaine
  - Mepivacaine
  - Prilocaine
  - Other__________
6. If using articaine, do you use it for all your dental procedures?
  - Yes
  - No
  - All except inferior alveolar nerve blocks
  - Other _____________
7. What is your current view of articaine in terms of safety and efficacy?
  - I am confident using articaine for all routine dental procedures
  - I am confident using articaine for some dental procedures
  - I am not confident using articaine for any dental procedures
  - Other __________
8. What currently influences for your perception of articaine in routine dental practice? (please click all applicable - multiple responses allowed)
  - Australian Dental Association guidelines
  - My country’s dental guidelines
  - My dental degree course
  - Ongoing continuing professional development courses
  - My own research
  - My dental colleagues
  - My mentor
  - Other _______
9. What are your views of articaine compared to lidocaine for use as a dental local anaesthetic for ALL routine dental procedures?
  - Articaine is as safe and efficacious as lidocaine
  - Articaine is as safe and more efficacious than lidocaine
  - Articaine is more efficacious than lidocaine, but not as safe
  - Articaine is not safe to use as a dental local anaesthetic
  - Other______________
10. Have you had any direct experience of your patients experiencing ongoing adverse reactions (>1 day) following administration of an inferior alveolar nerve block?
  - No
  - Yes (if yes please go to the next question)
11. If yes to the question 10, what anaesthetic was used for the IANB? Multiple answers possible: ___________
12. If yes to question 10, what type of adverse reaction was experienced by the patient after the inferior alveolar nerve block (please click all applicable - multiple responses allowed)
  - Paraesthesia (1)
  - Neuropathy (2)
  - Palsy (3)
  - Swelling, bruising (4)
  - Haematoma (5)
  - Palpitations, anxiety, shakes (6)
  - Syncope (7)
  - Vision changes (8)
  - Trismus (9)
  - Infection (10)
  - Other ____________ (11, plus new variable/value for text answer)
13. If yes to question 10, did you subsequently change your clinical practice?
  - No change to clinical practice
  - Continued using the LA, but not for all procedures
  - Stopped using that LA all together
  - Other __________
14. Is there any further information you would like to share about your personal experience with an LA-related adverse event? ___________________
